# Managing complications in patellar fracture surgery: tension band wiring challenges and innovations

**DOI:** 10.1097/JS9.0000000000001714

**Published:** 2024-06-04

**Authors:** Jie Peng, Mi Zou, Zhiqiang Qiu, Chen Li, Liang Hao

**Affiliations:** a Department of Orthopedics, The Second Affiliated Hospital; b The Second Clinical Medical College, Jiangxi Medical College, Nanchang University, Nanchang; c Department of Sports Medicine, Huashan Hospital, Fudan University, Shanghai, China

Highlights

In this manuscript, we address the significant challenges encountered in the management of patellar fractures, particularly focusing on the complications associated with tension band wiring (TBW) fixation and the innovative strategies proposed to mitigate these challenges. Our work highlights the following key points:

- Clinical significance: Patellar fractures are commonly treated with TBW fixation due to its advantages in reducing periosteal stripping, cost-effectiveness, and mature technical approach. However, potential complications such as wire breakage and migration can lead to severe consequences, necessitating a thorough understanding and effective management strategies.
- Uncommon complications: We shed light on the uncommon complication of intra-articular migration following wire breakage and the even rarer phenomenon of migration from intra-articular to extra-articular space. Through a detailed analysis of contributing factors and clinical cases, we emphasize the importance of early detection and intervention in preventing adverse outcomes.
- Innovative solutions: Our manuscript presents innovative alternatives to traditional TBW fixation, including percutaneous minimally invasive techniques, absorbable cannulated screws, and titanium cable fixation. These approaches offer potential advantages such as reduced soft tissue irritation, improved stability, and shorter operation times, contributing to enhanced patient outcomes.
- Clinical implications: We advocate for enhanced preoperative evaluation, personalized treatment planning, and diligent postoperative monitoring to optimize outcomes and minimize complications in patellar fracture surgery. Our recommendations emphasize the importance of a comprehensive approach to patient care.

We believe that our manuscript contributes valuable insights into the management of patellar fractures and offers practical guidance for orthopedic surgeons facing these challenges. The incorporation of clinical cases and innovative solutions underscores the relevance and applicability of our work to the broader surgical community.

*Dear Editor*,

Patellar fractures are commonly treated with tension band wiring (TBW). Tension band wiring fixation (TBWF) supports the healing process by maintaining the anatomical structure of the patella and enhancing tension within the surrounding soft tissues. Compared to other surgical strategies, this treatment method has significant advantages in reducing periosteal stripping, being cost-effective, and having a mature technical approach, leading to its widespread clinical application^1^. However, the potential complications of tension band wire broken and migration can result in severe consequences such as refracture migration, infection, nerve and vascular damage, joint stiffness and dysfunction, articular cartilage and joint capsule injuries, long-term pain and swelling, pericardial effusion, and pulmonary embolism.

Intra-articular migration following wire broken is uncommon, and the subsequent migration from intra-articular to extra-articular space is even rarer. In this scenario, as the patient’s range of motion increases, the widening of the fracture gap may lead to the wire entering the joint space through the anterior aspect of the patella along the pseudojoint line. Subsequently, the wire may migrate outside the joint space along muscles, tendons, or other soft tissues. The wire may also bypass the pseudojoint line and enter the joint space by passing through the joint capsule, along tendons or ligaments, or via synovial fluid. Previous reports have documented cases of patellar tension band wire broken leading to knee joint locking and migration of fractured Kirschner wires to the popliteal fossa and lateral foot^2^, even entering visceral organs through the circulatory system. During the migration process, the broken wire’s repeated friction with articular cartilage and synovium can lead to cartilage damage, synovial hyperplasia, and inflammation, resulting in joint swelling and pain. Factors contributing to wire broken and migration include material properties, stress distribution in the tension band, patient overactivity, chronic fatigue accumulation, joint swelling, and potential joint capsule damage.

In recent years, complications after tension band surgery for patellar fractures have received increasing attention, and some scholars have proposed some new feasible alternatives. For example, the percutaneous minimally invasive technology of cannulated screws combined with high-strength sutures and Nice knots has smaller incisions, reduces soft tissue irritation complications, and promotes functional recovery. Cannulated screws can also be replaced with absorbable cannulated screws (TBSAS)^3^, which can significantly shorten the operation time, improve safety and stability, and effectively reduce complications caused by metal internal fixation and trauma caused by secondary operations. In addition, the steel wire can be replaced by titanium cable, which can improve the stability of internal fixation, greatly reducing the incidence of non-union of fractures or wire broken, and using modified ‘8’ tension bands for fixation is more effective^4^. There are also some studies showing that percutaneous fixation of three cannulated screws (TCS), the use of Ding’s screws^5^, or three headless compression screws (HCS) in a posterior triangular configuration all show good biomechanical stability. Some new surgical techniques such as nickel titanium (NiTi) patellar concentrator (NT-PC), double suture cerclage reduction technology combined with Nice knot, double pulley technology combined with suture bridge technology and double row anchor suture bridge technology can shorten the operation time, which are very helpful for clinical practice. However, these alternative approaches, due to a lack of robust biomechanical and clinical trials, high costs, and relatively immature technology, have not yet replaced traditional methods.

We advocate for enhanced preoperative evaluation, personalized treatment planning for patients, careful consideration of potential complications, and diligent monitoring and follow-up postoperatively to improve outcomes following patellar fracture surgery and reduce complications. Given the uncertainty of wire broken and migration and the severity of associated complications, preoperative imaging localization, intraoperative fluoroscopic localization, and postoperative reimaging and follow-up are crucial. Early surgical removal should be performed upon detection of migration (Fig. 1).

**Figure 1:**
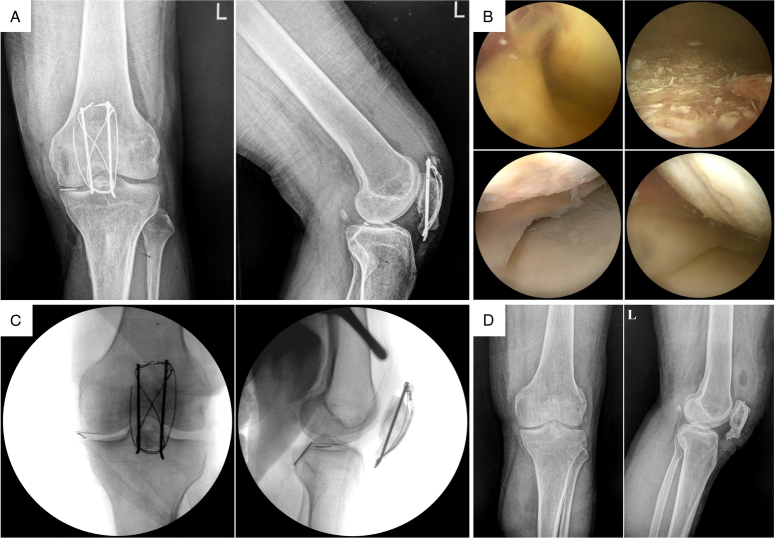
Series of X-ray images of a 43-year-old middle-aged male who underwent open reduction internal fixation surgery for left patellar fracture 7 years ago. He occasionally experienced discomfort postoperatively but did not adhere to regular follow-ups. Recently, he presented with 1-week persistent left knee swelling, pain, and limited mobility. X-ray examination revealed the broken of the patellar tension band wire, which had migrated from the anterior aspect of the patella into the joint space; intraoperatively, arthroscopic findings showed articular cartilage and joint capsule injuries, with no visible wire, but fluoroscopic localization revealed wire migration outside the joint space. The patient underwent arthroscopic and open surgical wire removal. Postoperatively, he received antimicrobial and pain relief medications. Follow-up appointments have been scheduled for ongoing monitoring. (A) Preoperative knee X-ray image showing patellar fracture with heterotopic ossification, multiple broken of the tension band wire, with displaced wire within the joint space. (B) Intraoperative arthroscopic image showing chondral injury on the medial femoral condyle, marked synovial hyperplasia in the suprapatellar pouch, and yellow-colored joint fluid. (C) Intraoperative fluoroscopic image of the knee joint shows that the wire foreign body has moved out of the joint cavity. (D) Postoperative knee X-ray image showing complete removal of the broken wire.

## Ethical approval

We have obtained ethical approval and consent from The Biomedical Research Ethics Committee of the Second Affiliated Hospital of Nanchang University (IIT-O-2024-113).

## Consent

We have had the patient’s written consent for publication.

## Source of funding

Not applicable.

## Author contribution

J.P. and M.Z. wrote this original manuscript, Z.Q. provided imaging data, C.L. and L.H. jointly participated in the patient’s surgery, and L.H. reviewed and revised this manuscript. Everyone approved the publication of this manuscript.

## Conflicts of interest disclosure

We have read and understood the policy on declaration of interests and declare no competing financial interests.

## Research registration unique identifying number (UIN)

This is a letter to editor and the registry is not applicable.

## Guarantor

The Guarantor is Liang Hao.

## Data availability statement

Not applicable.

## Provenance and peer review

Not applicable.
